# The Production, Efficacy, and Safety of Machine-Generated Bicarbonate Solution for Continuous Venovenous Hemodialysis (CVVHD): The Cleveland Clinic Method

**DOI:** 10.1016/j.xkme.2021.01.003

**Published:** 2021-03-10

**Authors:** Jonathan J. Taliercio, Georges Nakhoul, Tushar J. Vachharajani, Matthew Layne, John Sedor, George Thomas, Ali Mehdi, Robert Heyka, Sevag Demirjian

**Affiliations:** Department of Nephrology and Hypertension, Glickman Urological and Kidney Institute, Cleveland Clinic Lerner College of Medicine of Case Western Reserve University, Cleveland Clinic, Cleveland, OH

**Keywords:** AKI, homemade dialysate, CVVHD, CRRT, continuous veno-venous hemodialysis, machine generated bicarbonate, ultrapure, acute renal failure, hemodialysis, end stage kidney disease

## Abstract

**Rationale & Objective:**

Since 1994, the Nephrology and Hypertension Department at the Cleveland Clinic has prepared and used bicarbonate-based solution for continuous venovenous hemodialysis (CVVHD) using a standard volumetric hemodialysis machine rather than purchasing from a commercial vendor. This report describes the process of producing Cleveland Clinic UltraPure Solution (CCUPS), quality and safety monitoring, economic costs, and clinical outcomes.

**Study Design:**

Retrospective study.

**Setting & Participants:**

CVVHD experience at Cleveland Clinic, focusing on dialysate production, institutional factors, and patients requiring continuous kidney replacement therapy. Production is shown at www.youtube.com/watch?v=WGQgephMEwA.

**Outcomes:**

Feasibility, safety , and cost.

**Results:**

Of 6,426 patients treated between 2011 and 2019 with continuous kidney replacement therapy, 59% were men, 71% were White, 40% had diabetes mellitus, and 74% presented with acute kidney injury. 98% of patients were treated with CVVHD using CCUPS, while the remaining 2% were treated with either continuous venovenous hemofiltration or continuous venovenous hemodiafiltration using commercial solution. The prescribed and delivered effluent doses were 24.8 (IQR) versus 20.7 mL/kg/h (IQR), respectively. CCUPS was as effective in restoring electrolyte and serum bicarbonate levels and reducing phosphate, creatinine, and serum urea nitrogen levels as compared with packaged commercial solution over a 3-day period following initiation of dialysis, with a comparable effluent dose. Among those with acute kidney injury, mortality was similar to that predicted with the 60-day acute kidney injury predicted mortality score (r = 0.997; CI: 0.989-0.999). At our institution, the cost of production for 1 L of CCUPS is $0.67, which is considerably less than the cost of commercially purchased fluid.

**Limitations:**

Observational design without a rigorous control group.

**Conclusions:**

CVVHD using locally generated dialysate is safe and cost-effective.

## Graphical abstract

**Figure:**
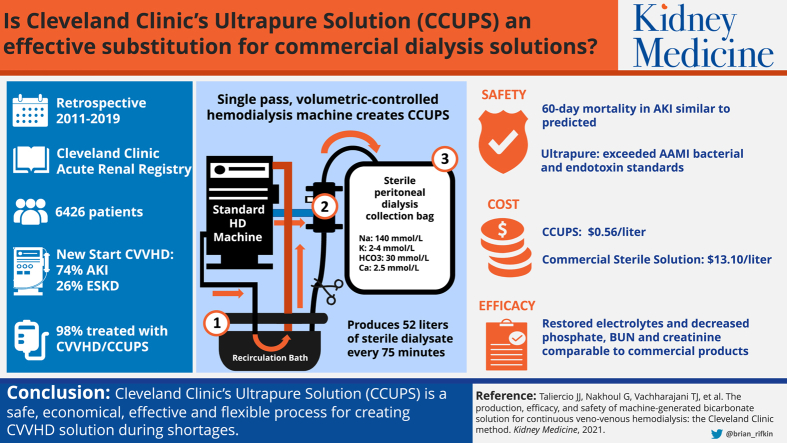

**Plain-Language Summary**

We describe the Cleveland Clinic’s vast experience with the in-house production, safety, and clinical efficacy of ultrapure dialysate for patients receiving continuous venovenous hemodialysis. We believe that this is a timely article in the coronavirus disease 2019 era as global dialysate and supply chains are disrupted. Many nephrologists and health care institutions have had to provide alternative modes of therapy and ration dialysis due to shortages. We hope to share our 25-year experience so that other institutions may consider adopting our practice.

Editorial, p. 321

The coronavirus disease 2019 pandemic has led to disruptions within the global supply chains and scarcity in medical supplies such as personal protective equipment and ventilators.^1^ Dialysis equipment, supplies, and personnel are not immune to this problem, especially in hard-hit areas such as New York City.^2^, ^3^, ^4^ Depending on acute kidney injury (AKI) definitions and staging reported, the incidence of AKI in hospitalized patients infected with SARS-CoV-2 ranges from 5% to 29%.^5^, ^6^, ^7^, ^8^, ^9^ Moreover, the high-risk end-stage kidney disease (ESKD) population is particularly vulnerable, with a pre-pandemic track record of twice-a-year hospitalizations and a 35% readmission rate within 30 days.^10^

Beyond the sheer number of hospitalized patients with AKI and ESKD requiring kidney replacement therapy (KRT), frequent therapy to optimize volume is often indicated in patients with acute lung injury and acute respiratory disease. To manage resources during a dialysis surge, caregivers have had to ration dialysis dose, delay initiation, or withhold therapy in some patients.^11^ A major driver in the supply shortage affecting the provision of continuous KRT (CKRT) in critically ill patients is the current and projected shortages in ready-to-use commercially available dialysis fluids.^11^ The Cleveland Clinic Nephrology and Hypertension Department has more than 25 years’ experience in the production and clinical use of machine-generated bicarbonate dialysate for continuous venovenous hemodialysis (CVVHD) and has been approached by many nephrologists at other institutions inquiring about the methodology.^12^^,^^13^ The purpose of this report is to describe the production, efficacy, and safety of the Cleveland Clinic UltraPure Solution (CCUPS) for CVVHD.

## Methods

### Study Design and Participants

In the current retrospective study, we included all adult patients hospitalized at Cleveland Clinic intensive care units from August 2011 to October 2019 who required CKRT. Patients with ESKD were included in the analysis. Informed consent was waived because all information was deidentified, there was no more than minimal risk to the patients, and the waiver will not adversely affect the rights and welfare of the patients.

### Data Collection

We used the Cleveland Clinic Acute Renal Registry to extract demographic, clinical, laboratory, dialysis, and outcome information. The Cleveland Clinic Acute Renal Registry, established in 1987, and the current retrospective study were approved by the Cleveland Clinic Institutional Review Board (approval number 5000).

### Missing Variables

Fifty-two patients with missing data regarding type of solution used were excluded from analysis. Metabolic panel components were missing in 126 or fewer patients. Serum phosphate level was missing in 744 patients, and magnesium level, in 635 patients, before dialysis initiation. We presented nonimputed results; however, multiple imputations with chained equations did not alter our findings.

### Statistical Analysis

We presented all continuous variables as median with interquartile range (IQR), and categorical variables, as count with percentage. We used repeated mixed regression models to assess change in laboratory levels at dialysis initiation and follow-up days. In the subgroup of patients with AKI, we compared predicted hospital mortality per Demirjian scoring model with that observed in our study.^14^

### CCUPS Preparation

We prepare CCUPS using a volumetric-controlled single-pass hemodialysis machine (Fig S1). The machine proportions and mixes 2 concentrates (acid and bicarbonate) with heated ultrapure water (produced by reverse osmosis) to create dialysate. The solution is a replication of the dialysate used in intermittent hemodialysis with the corresponding electrolyte bath. The required additional step is back-filtering the solution from the dialysate compartment of the filter to the blood compartment and subsequent collection in a sterile bag. Our default dialysate settings are the following concentrations: sodium, 140 mmol/L; bicarbonate, 30 mmol/L; calcium, 2.5 mmol/L; and potassium in the range of 2 to 4 mmol/L, although periodically, custom composition of varying electrolyte concentrations are produced in specific clinical situations such as severe hyponatremia.

### Connectology

We connect the dialysate feed line to one end of the dialyzer ports, while the unused port is capped (Fig S2). Next, we place the return dialysate line in a container of replenishable water to complete a closed dialysate circuit. We inflow the dialysate at 800 mL/min into the dialysate compartment of a high-flux hollow-fiber polysulfone membrane dialyzer and allow the transfer by back-filtration from the dialysate compartment into the dialyzer "blood" compartment through sterile lines and a splitter, from which it is collected into sterile (6.5-L) bags. We feed the nondraining side of the dialyzer blood compartment to a sterile line that we clamp during the procedure. For comprehensive step-by-step instructions on the production of CCUPS, we refer you to the following instructional video: www.youtube.com/watch?v=WGQgephMEwA.

### Quality and Safety

We use a double-pass reverse-osmosis machine (BioPureHX2) to produce product water that is passed through a 0.02-μm endotoxin microfilter in addition to the filter equipped on the dialysis machine in the path of dialysate fluid after addition of the base and acid concentrates. We perform daily heat and weekly chemical disinfection of the dialysis machine with extended cycles. We perform weekly heat disinfection of the entire water system with monthly monitoring of the loop and designated dialysate machines. The bags are stored for a maximum of 5 days at room temperature. Last year we produced 116 bags (754 L/d) of CUPPS on average a day. However, the quantity of produced dialysate varies day to day based on projected needs and rarely stays in storage more than 1 to 2 days due to the first-in first-out bag consumption protocol.

The hemodialysis solution produced is “ultrapure” because it exceeds the American Association of Medical Instrumentation (AAMI) standards for annual testing of bacterial (<0.1 colony-forming unit [CFU]/mL) and endotoxin unit (EU/mL < 0.03) limits (revised in 2015).^15^ We perform more rigorous monthly testing of the dialysate during production and sample stored fluid on different days to assess quality to confirm that bacterial colony counts and endotoxin levels ensure that ultrapure dialysis fluid standards are maintained (Fig S3). All water tests are sent to an independent third-party laboratory to analyze and verify the results. To substantiate our claim of the production of ultrapure dialysate, the third party uses the membrane filtration culture method (9215D), in which a large volume of the fluid tested is passed through a sterile filter, which is then cultured. This method is used in other parts of the world where ultrapure dialysate is a standard.^16^ The laboratory has a lower limit of 0.01 CFU/mL and 0.001 EU/mL, which is well below AAMI ultrapure standards. See Table 1 for an abridged historical reference of our water testing results. The electrolyte concentration is confirmed by using the dialysate machine’s conductivity meter during the time of production. In addition, conductivity and pH of the dialysate are verified using a pHoenix meter during the time of hemodialysis production. Periodic sampling of the stored dialysate may be used if clinically required or desired to determine quality and safety.

**Table 1:** Quarterly Machine and Solution Testing for Endotoxins and Cultures at Cleveland Clinic

Date	Machine 1, CFU/mL	Machine 1, EU/mL	Machine 2, CFU/mLl	Machine 2, EU/mL	Bag 1, CFU/mL	Bag 1, EU/mL	Bag 2, CFU/mL	Bag 2, EU/mL
10/2020	0	0	0	0	0	0	0	0
06/2020	0	0	0	0	0	0	0	0
03/2020	0	0	0	0	0	0	0	0
01/2020	0	0	0	0	0	0	0	0
10/2019	0	0	0	0	0	0	0	0
06/2019	0	0	0	0	0	0	0	0
03/2019	0	0	0	0	0	0	0	0
01/2019	0	0	0	0	0	0	0	0
10/2018	0	0	0	0	0	0	0	0
06/2018	0	0	0	0	0	0	0	0
03/2018	0	0	0	0	0	0	0	0
01/2018	0	0	0	0	0	0	0	0
10/2017	0	0	0	0	0	0	0	0
06/2017	0	0	0	0	0	0	0	0
03/2017	0	0	0	0	0	0	0	0
01/2017	0	0	0	0	0	0	0	0

All results from a detection limit of 0.01 CFU/mL and 0.001 EU/mL.

Abbreviations: CFU, colony-forming unit; EU, endotoxin unit.

## Results

### Cohort Description

We performed 6,426 new CKRT starts from August 2011 to October 2019, with most (98%) being CVVHD using CCUPS. The remaining 2% of CKRT were either continuous venovenous hemofiltration (CVVHF) or continuous venovenous hemodiafiltration using commercial sterile solution. Seventy-four percent of patients started were patients with AKI, whereas the remaining 26% were patients with ESKD. The median age was 63 years, 59% were men, and 40% had diabetes mellitus. Patients' serum urea nitrogen and serum creatinine levels on initiation of dialysis were 57 mg/dL and 3.59 mg/dL, respectively. Additionally, patients had elevated phosphate levels, with a median level of 5.5 (IQR, 2.9) mg/dL, median potassium level of 4.5 (IQR, 1.1) mmol/L, and median bicarbonate level of 20 (IQR, 8) mmol/L (Table 2).

**Table 2:** Baseline Clinical Characteristics and Laboratory at Continuous Dialysis Initiation

Variable	All Patients
Age, y	63 (18)
Male sex	3,766 (59%)
White race	4,534 (71%)
Weight, kg	94.7 (33.2)
Hypertension	4,209 (65%)
Diabetes mellitus	2,580 (40%)
Chronic obstructive lung disease	1,438 (22%)
Congestive heart failure	1,753 (27%)
Coronary arterial disease	1,587 (25%)
End-stage kidney disease	1,660 (26%)
Dialysis solution type	
Machine generated	6,319 (98%)
Commercial	107 (2%)
Effluent rate post day 1	
Prescribed, mL/kg/h	24.8 (5.6)
Delivered, mL/kg/h	20.7 (8)
Laboratory at dialysis initiation:	
Creatinine, mg/dL	3.59 (2.55)
Serum urea nitrogen, mg/dL	57 (50)
Sodium, mmol/dL	136 (7)
Bicarbonate, mmol/dL	20 (8)
Potassium, mmol/L	4.5 (1.1)
Calcium, mg/dL	8.2 (1.2)
Phosphate, mg/dL	5.5 (2.9)
Magnesium, mg/dL	2.2 (0.3)
Lactate, mmol/L	2.1 (3.5)

N = 6,426. Values expressed as number (percent) or median (interquartile range).

### Efficacy and Safety

**Figure 1:**
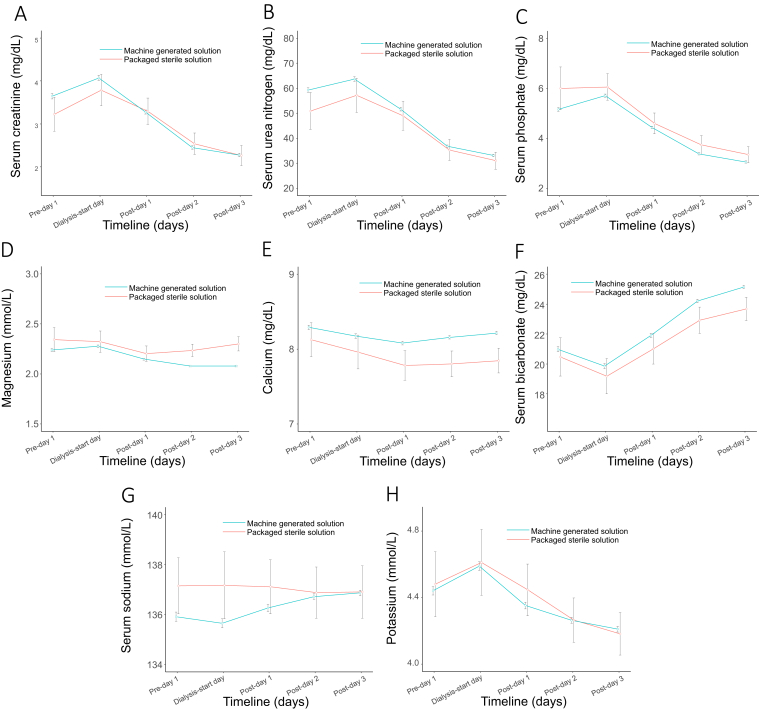
Effect of type of solution on solute trajectories in the peridialysis period with 95% CIs. (A) Serum creatinine, (B) serum urea nitrogen, (C) serum phosphate, (D) serum magnesium, (E) serum calcium, (F) serum bicarbonate, (G) serum sodium, and (H) serum potassium.

**Figure 2:**
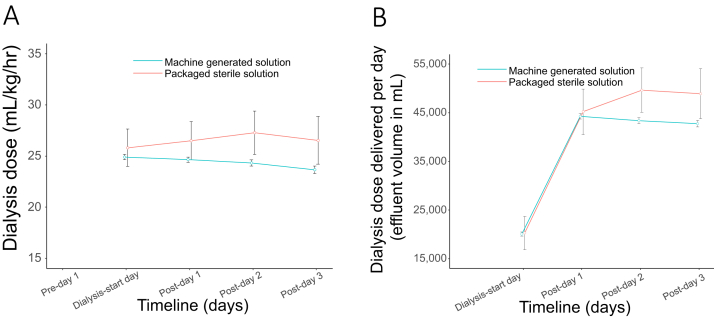
Effluent dose delivered in the first 96 hours of dialysis initiation. (A) Effluent rate per weight per hour (mL) and (B) effluent volume delivered per day (mL).

**Figure 3:**
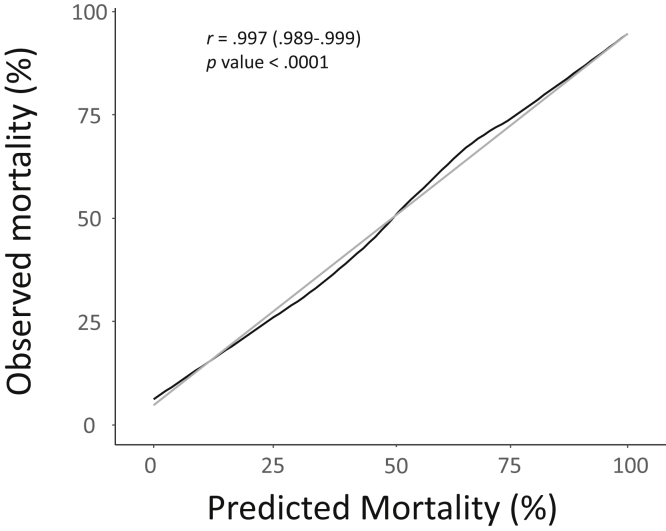
Observed versus predicted mortality per acute kidney injury (AKI)-specific scoring system. Black line denotes observed mortality and gray line denotes predicted mortality per mortality predictive model in patients with AKI treated with Cleveland Clinic UltraPure Solution.^14^ Cost of ultrapure dialysate production per liter for 2019.

The prescribed effluent rate was 24.8 (IQR, 5.6) mL/kg/h, resulting in a median volume of dose delivered of 20.7 (IQR, 8) mL/kg/h after accounting for machine downtime. CCUPS was as effective in restoring electrolyte and serum bicarbonate levels and reducing phosphate, creatinine, and serum urea nitrogen levels as compared with packaged commercial solution (Fig 1), with similar effluent rates (Fig 2). In mixed linear regression analyses in which solute levels were used as the dependent variable; all models showed changes in levels starting on dialysis day to post day 3. During the course of this 8-year period, which encompassed approximately 96 monthly water checks, on 1 occasion the monthly dialysis machine tested positive for bacteria (75 and 82 CFU/mL). Ultimately, this was deemed to be contamination related to acquisition and/or processing; repeat testing of the machine and bags of the same batch returned at 0 CFU/mL before any disinfecting interventions. We compared 60-day AKI predicted mortality score with the observed mortality in the subgroup with AKI,^14^ which showed similar results (Fig 3).

### Cost

At a flow rate of 800 mL/min, 1 intermittent dialysis machine has the capacity to produce eight 6.5-L bags in 75 minutes. A single machine can produce a maximum of 112 bags per day (12 batches × 8 bags) when accounting for 3 hours of machine disinfection time and 15 minutes of set-up (labor) time per batch. We operate 2 machines simultaneously to limit CCUPS production to daytime and weekdays only.

The largest cost components in the machine-generated solution production are the sterile bag ($2.08) in disposables and labor ($0.92) used for the process: these represent 59% and 26% of the total cost of a single 6.5-L bag produced ($3.62), respectively. The daily fixed expenses of consumables and maintenance remain minimal if the number of bags produced are close to capacity (Table 3). Because the actual cost of CKRT solution is proprietary to individual institutions, we compared the average wholesale price of commercial nonsterile and sterile products, which is $10.50/L and $13.10/L, respectively.^17^^,^^18^ CCUPS is 19-fold ($0.56 vs $10.50 per L) and 23-fold ($0.56 vs $13.10) less expensive, respectively (Table 4).

**Table 3:** Cost of Ultrapure Dialysate Production Per Liter for 2019

Item	Per Liter
Machine related	
Dialysis machine	$0.00799
Disinfection^a^	$0.00092
Laboratory	$0.00672
Maintenance^b^	$0.00591
Y piece	$0.00938
Straight line	$0.00998
Dialyzer	$0.01572
Solution related:	
Reverse-osmosis water	$0.00630
Bicarbonate	$0.00634
Acid	$0.01133
Sterile bag	$0.31958
Label	$0.00583
Zip ties	$0.00919
Employee labor^c^	$0.14103
Total:	$0.55622

a Disinfection includes water, bicarbonate systems, and machine disinfection.

b Dialysis parts and repair.

c Fifteen minutes' labor per batch (8 bags).

**Table 4:** Continuous Dialysis Solution Cost Comparison Based on Product^a^

Volume^a^	Cleveland Clinic Method	Nonsterile Commercial	Sterile Commercial Fluid
1 L	$0.56	$10.50	$13.10
275,360 L^b^	$154,202	$2,891,280	$3,607,216

a Comparison does not include costs due to pharmacy storage and dispensation associated with commercial solutions.

b Total amount of Cleveland Clinic Acute Renal Registry produced in 2019 at Cleveland Clinic.

## Discussion

The current report describes an update on the clinical application of the Cleveland Clinic method for large-scale machine-generated bicarbonate solution production. We describe the connectology using repurposed sterile medical-grade readily available components, the biochemical profile in a large and longitudinal cohort compared with sterile commercial solution, safety through updated quality assurance and mortality outcomes, and economic savings considering fixed and variable costs. In addition, the Cleveland Clinic method has the advantage of being surge proof, considering the projected shortage of commercial solutions, and can be used as a backup in emergency preparedness in every hospital dependent on CKRT.

The Cleveland Clinic method for machine-generated bicarbonate solution production is based on the well-established practice of dialysate solution production from treated municipal water supply and in-machine 3-way mixing with acid/base concentrates. The novelty of CCUPS is the brief storage of machine-generated dialysate using aseptic techniques for the subsequent use in CVVHD (diffusion-based dialysis only). We do not use CUPPS in convection-based therapies such as CVVHF as a replacement fluid. Replacement fluid used in convection-base therapies is required to be sterile because they are infused directly into patients and therefore designated as a “drug” by the US Food and Drug Administration.^19^

In accordance to these factors, we use machine-generated ultrapure bicarbonate solutions only in hemodialysis mode at Cleveland Clinic and resort to commercially available sterile solutions in hemofiltration and diafiltration. An alternative to commercial solution and CCUPS is pharmacy compounding of sterile solution. However, it is time-intensive, expensive, prone to human errors, and not amenable to large-scale production.

Detailed standard operating procedures and quality assurance programs are essential to ensure safety and enhance patient care. Improving dialysis machine technology and more stringent AAMI guidelines in dialysate preparation in fluid quality, water treatment equipment, and monitoring has led to increased purity of CCUPS. Five years ago, we raised our pyrogen and bacterial contamination thresholds to ultrapure dialysate standards and upgraded our main water treatment facility to a double-pass reverse-osmosis system. In a double-pass system, the permeate from the first pass becomes the feed water in the second pass, resulting in higher quality final water because it essentially passes through 2 reverse-osmosis systems. Although a portable reverse-osmosis machine may be used, the quality of the dialysate produced will depend on your water source.

In addition to microbiological monitoring, the implementation of disinfection procedures and filter changes is equally important. We also strongly recommend having a dialysis machine dedicated for bag production (not used for direct patient care) and equipped with ultrafilters installed in series to ensure high-quality dialysate (postmixing with concentrates). We also recommend a conservative shelf life of no longer than 5 days to maintain the integrity of the bag chemistry and avoid the potential of divalent micro- and macrocrystalization, neither of which we have experienced at this recommended shelf life. If microscopic crystals formed, they would be prevented from entering the patient by the CVVHD dialyzer filter. The concern with longer storage is divalent ion precipitation, such as calcium carbonate. The presence of acetic acid in the acid concentrate during mixing helps lower the pH of the dialysate, which in turn would prevent the precipitation of insoluble calcium and magnesium salts (as long as the stored bicarbonate solution does not lose carbon dioxide gas to become carbonate over time). Corradi et al similarly produced online-prepared hemodiafiltration fluid and demonstrated stable clinical electrolyte composition (sodium, potassium, chloride, and calcium), did not identify macrocrystalization (despite changes in pH and Pco_2_), and had negative fluid cultures, endotoxin, and molecular testing after 7 days of storage.^20^

The major clinical advantage of CCUPS is the reliable, on-demand, and scalable production of variable-composition solution without the need to resort to pharmacy compounding (which introduces cost and potential for human error). The latter provides the flexibility of treating acute dysnatremias and challenging acid-base disorders with custom-made dialysis solutions in a challenging critical care environment. As expected, machine-generated ultrapure bicarbonate solution had a similar impact on small-solute clearances compared with commercial sterile solution. Moreover, the 60-day observed mortality was in line with predicted mortality per a AKI-specific multicenter-derived severity score.^14^

One of the biggest obstacles facing widespread adoption of CKRT is the expense, of which commercial dialysis solution represents a large portion of the overall cost (Table 4). In our institution, the total median effluent volume used is 45 L/d, which costs $25.20 per day ($0.56/L × 45 L/d) to produce; the equivalent daily cost for commercial nonsterile solution is $472.50 ($10.50/L × 45 L/d) and commercial sterile solution is $589.50 ($13.10/L × 45 L/d). In 2019, we produced 275,360 L of CCUPS for a total cost of $154,202. Assuming average wholesale pricing, the same quantity of commercial nonsterile fluid would have cost the hospital $2,891,280, resulting in an annual savings of $2,737,078. Had our practice patterns been to solely prescribe CVVHF, using average wholesale prices, the cost to the system would have been $3,607,216 last year. Actual negotiated CKRT solution prices will be lower with larger discounts based on bigger volume purchases; however, CUPPS is considerably less expensive due to economics of scale.

A major limitation of the machine-generated dialysate is its restricted use to diffusion-based therapy. In our facility, we resort to commercial sterile fluid if hemofiltration or hemodiafiltration is prescribed per the attending nephrologist’s discretion, to be used as replacement fluid. However, to date, there have been no clinical outcome studies that favor convection versus diffusion-based modalities.^21^^,^^22^

In conclusion, the Cleveland Clinic method of machine-generated ultrapure dialysate production has been refined during the last 3 decades and remains a cost-effective, safe, efficacious, flexible, scalable, and easily adoptable process. We recommend that all large medical centers should consider alternative means of solution production or dialysis provision in the intensive care unit that bypasses predictable shortages in the supply chain as part of emergency preparedness.
