# Supplementary material for: The Production, Efficacy, and Safety of Machine-Generated Bicarbonate Solution for Continuous Venovenous Hemodialysis (CVVHD): The Cleveland Clinic Method
Source: Kidney Med. 2021 Mar 10;3(3):353–359.e1. doi: 10.1016/j.xkme.2021.01.003 (PMC8178460; doi:10.1016/j.xkme.2021.01.003)

Figure S1. Dialysate Qd 800 ml/min into a polysulphone dialyzer with back-filtration through sterile lines and a splitter from which it is collected into sterile (6.5 Liter) peritoneal bags.

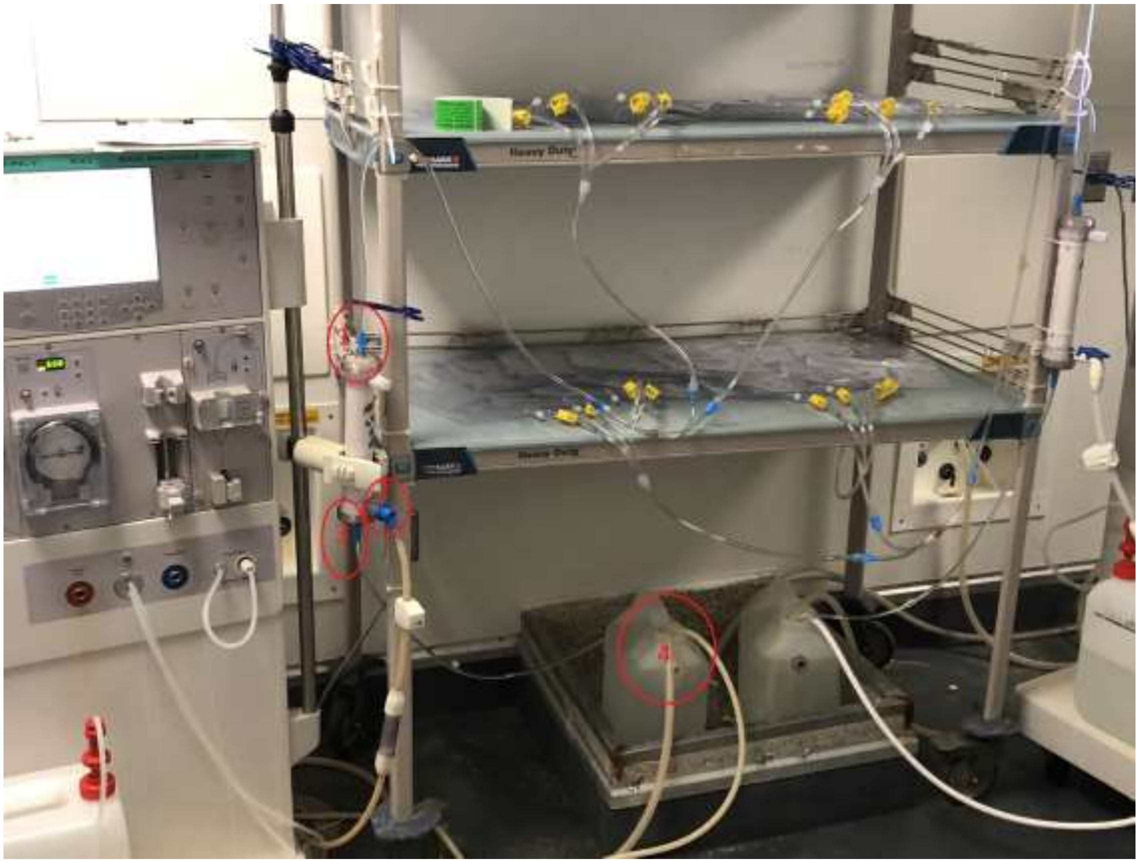

Figure S2. Back-filtration of dialysate through a polysulphone dialyzer into the "blood compartment" with the other dialysate port capped.

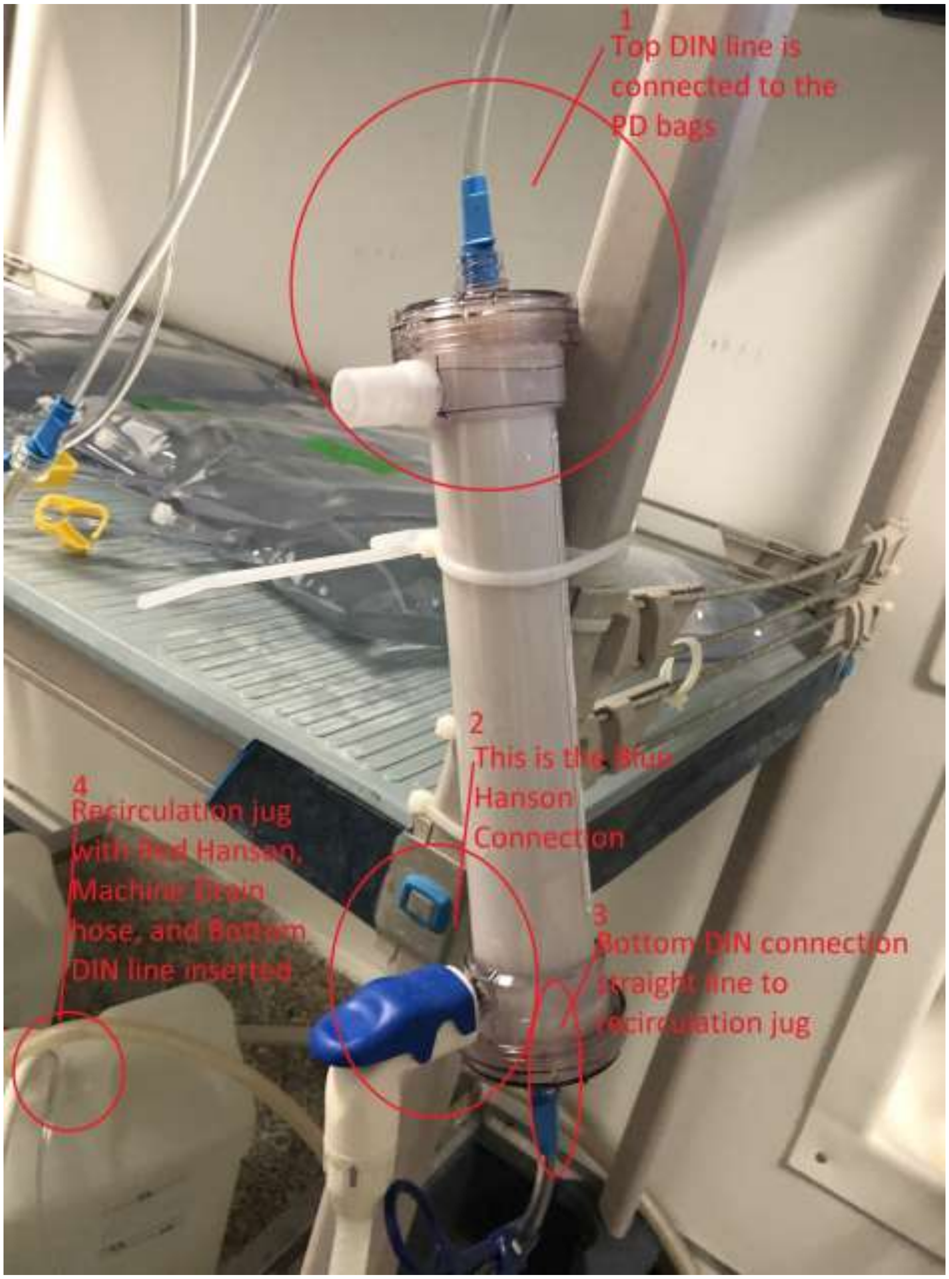

Figure S3. Select electrolyte composition of two representative bags tested on day of production, day three and day five.

A. Sodium concentration in repeated measures in two bag samples (dashed line represents machine predicted concentration)

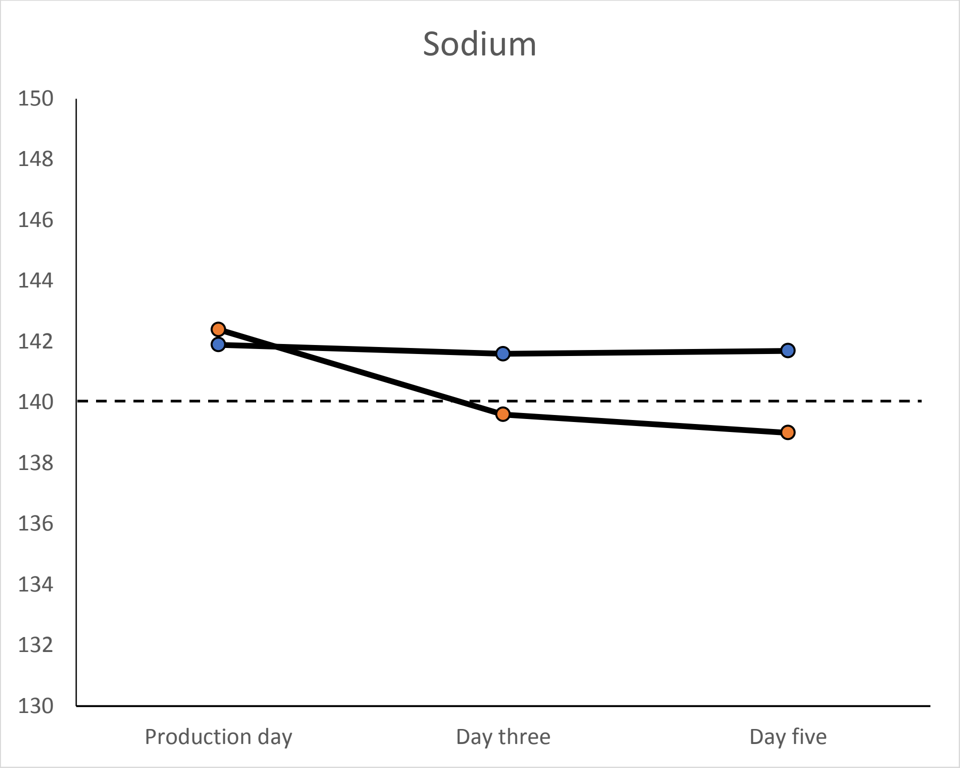

B. Potassium concentration in repeated measures in two bag samples (dashed line represents machine predicted concentration)

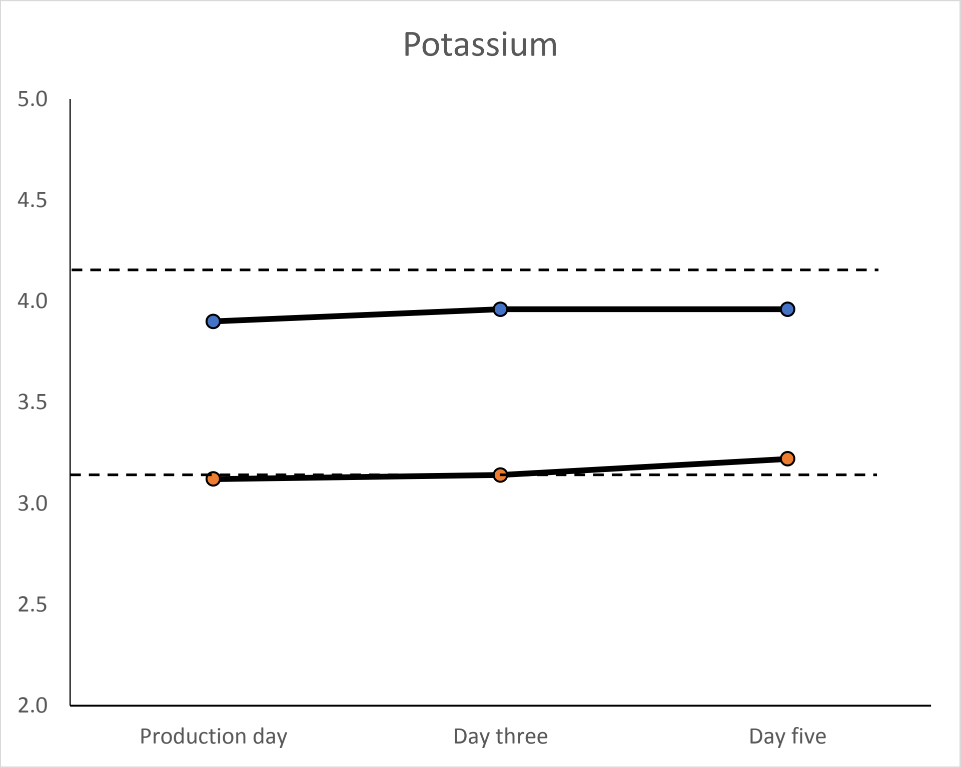

C. Chloride concentration in repeated measures in two bag samples (dashed line represents machine predicted concentration)

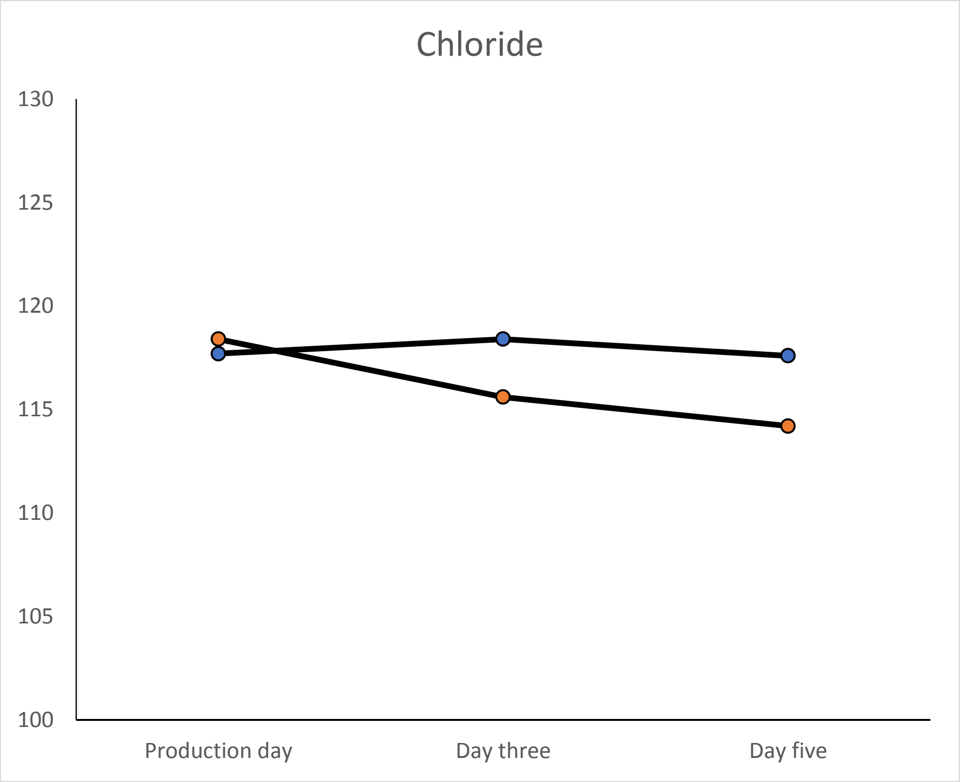

D. Bicarbonate concentration in repeated measures in two bag samples (dashed line represents machine predicted concentration)

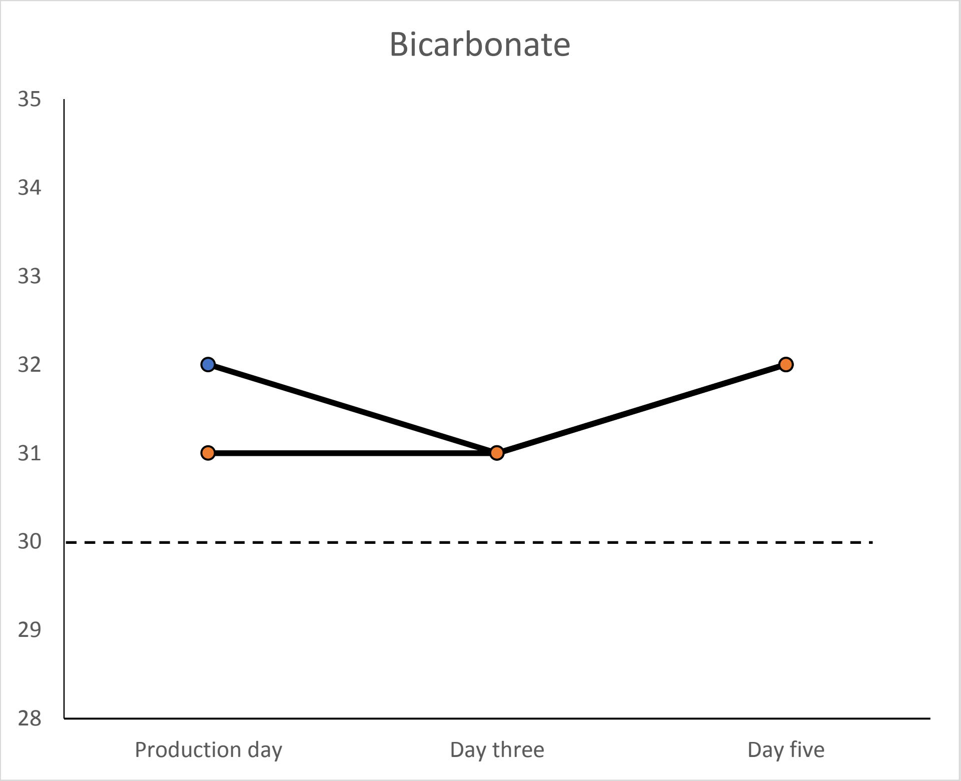

E. Calcium concentration in repeated measures in two bag samples (dashed line represents machine predicted concentration)

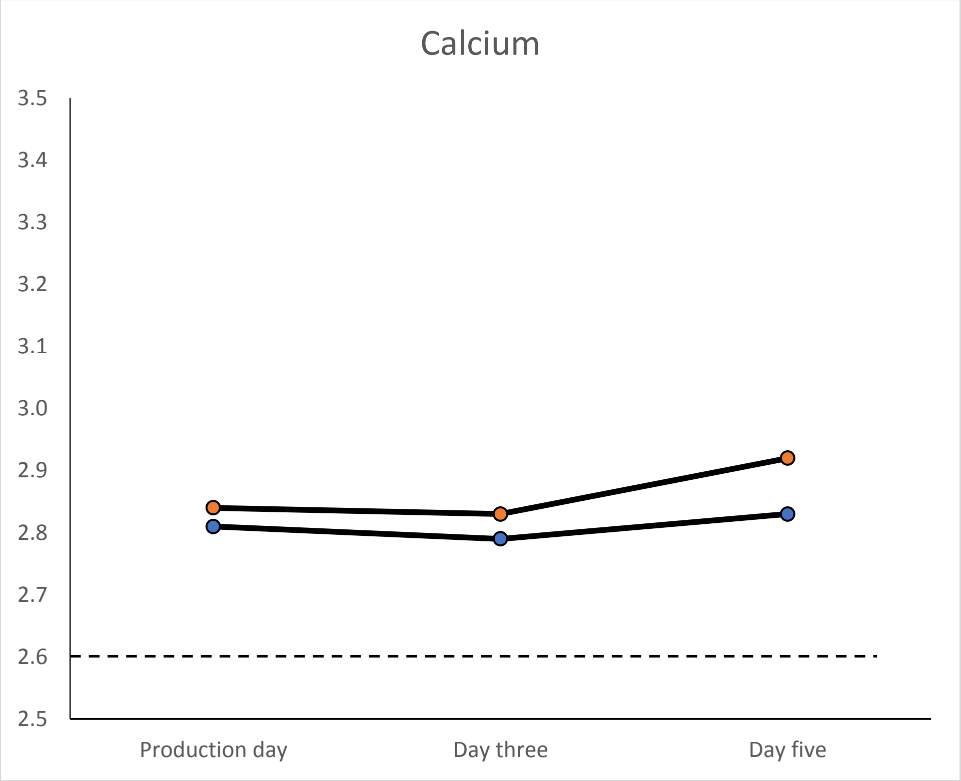

F. Magnesium concentration in repeated measures in two bag samples (dashed line represents machine predicted concentration)

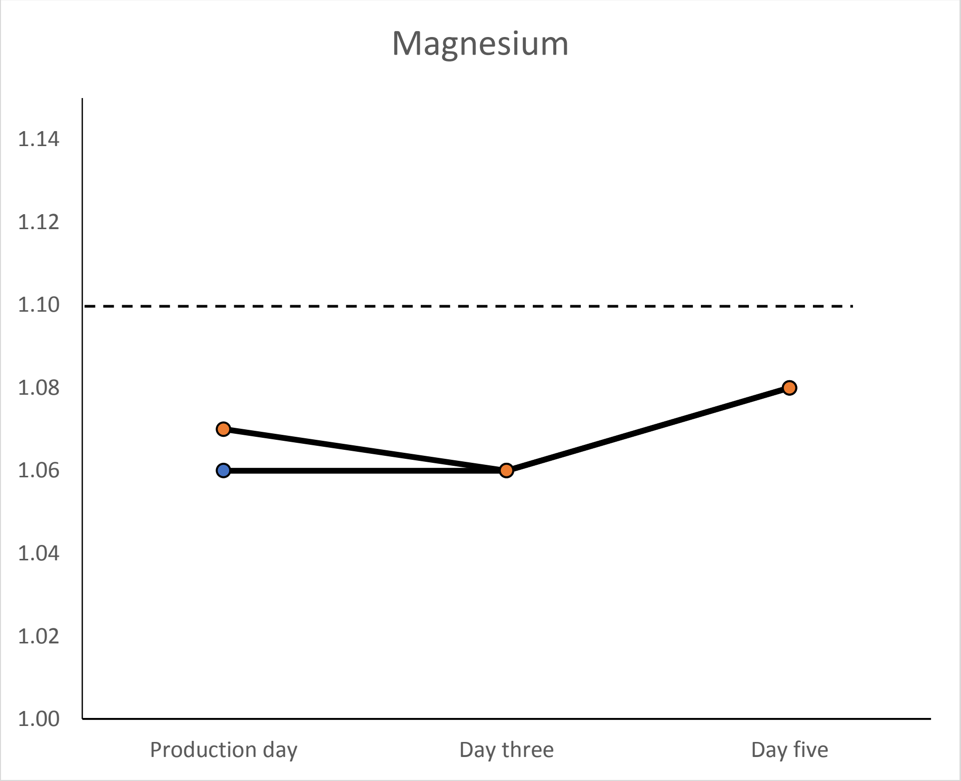

Supplement: Supplementary File (PDF) — Figures S1-S3. [file mmc1.pdf]
